# Application of the SCAI classification to admission of patients with cardiogenic shock: Analysis of a tertiary care center in a middle-income country

**DOI:** 10.1371/journal.pone.0273086

**Published:** 2022-08-16

**Authors:** Héctor González-Pacheco, Rodrigo Gopar-Nieto, Diego Araiza-Garaygordobil, José Luis Briseño-Cruz, Guering Eid-Lidt, Jorge Arturo Ortega-Hernandez, Daniel Sierra-Lara, Alfredo Altamirano-Castillo, Salvador Mendoza-García, Daniel Manzur-Sandoval, Klayder Melissa Aguilar-Montaño, Heriberto Ontiveros-Mercado, Jorge Iván García-Espinosa, Pablo Esteban Pérez-Pinetta, Alexandra Arias-Mendoza

**Affiliations:** 1 Coronary Care Unit, National Institute of Cardiology in Mexico City, Mexico City, Mexico; 2 Department of Interventional Cardiology, National Institute of Cardiology in Mexico City, Mexico City, Mexico; University of Florida, UNITED STATES

## Abstract

**Aims:**

The Society of Cardiovascular Angiography and Interventions (SCAI) shock stages have been applied and validated in high-income countries with access to advanced therapies. We applied the SCAI scheme at the time of admission in order to improve the risk stratification for 30-day mortality in a retrospective cohort of patients with STEMI in a middle-income country hospital at admission.

**Methods:**

This is a retrospective cohort study, we analyzed 7,143 ST-segment elevation myocardial infarction (STEMI) patients. At admission, patients were stratified by the SCAI shock stages. Multivariate analysis was used to assess the association between SCAI shock stages to 30-day mortality.

**Results:**

The distribution of the patients across SCAI shock stages was 82.2%, 9.3%, 1.2%, 1.5%, and 0.8% to A, B, C, D, and E, respectively. Patients with SCAI stages C, D, and E were more likely to have high-risk features. There was a stepwise significant increase in unadjusted 30-day mortality across the SCAI shock stages (6.3%, 8.4%, 62.4%, 75.2% and 88.3% for A, B, C, D and E, respectively; P < 0.0001, C-statistic, 0.64). A trend toward a lower 30-day survival probability was observed in the patients with advanced CS (30.3, 15.4%, and 8.3%, SCAI shock stages C, D, and E, respectively, Log-rank P-value <0.0001). After multivariable adjustment, SCAI shock stages C, D, and E were independently associated with an increased risk of 30-day death (hazard ratio 1.42 [P = 0.02], 2.30 [P<0.0001], and 3.44 [P<0.0001], respectively).

**Conclusion:**

The SCAI shock stages applied in patients con STEMI at the time of admission, is a useful tool for risk stratification in patients across the full spectrum of CS and is a predictor of 30-day mortality.

## Introduction

Cardiogenic shock (CS) is defined as a severe impairment of myocardial performance that results in diminished cardiac output, end-organ hypoperfusion, and tissue hypoxia. Acute myocardial infarction (AMI) accounts for roughly 80% of patients in CS [1]. ST-segment-elevation myocardial infarction (STEMI) is associated with a higher risk of developing CS and is considered the leading cause of death in these patients [2]. Despite improvements in the management of CS, in-hospital mortality remains high (27%–51%) [3]. Over time, some CS classifications had been developed, however classifying CS remains a challenge, largely due to the wide spectrum of clinical severity among patients presenting with this condition. A shock classification scheme was recently proposed by the Society for Cardiovascular Angiography and Interventions (SCAI) [4]. The purpose of this classification is to facilitate CS patient care and related research by providing a uniform system to identify patients with an increased risk of dying, based on hemodynamic parameters, indicators of tissue perfusion, and response to initial interventions. This system includes five stages of CS: (A) at risk for CS, (B) beginning CS, (C) classic CS, (D) deteriorating CS, and (E) extreme CS.

There have been several validation studies performed in the United States and Germany, they have shown the correlation of SCAI shock stages with mortality in different clinical subgroups of patients with CS, including CS with and without AMI, they also have showed that the SCAI clasification is a strong predictor of mortality [5–7].

At this time, there is little information about application of the SCAI classification at admission to a Coronary Care Unit (CCU) in a middle-income country to predict adverse events in patients with STEMI. Therefore, the primary aim of this study was to apply the SCAI scheme at the time of admission and its prognostic implication in each of the stages of the classification in order to improve the risk stratification for 30-day mortality in a retrospective cohort of patients hospitalized for STEMI.

## Materials and methods

This was a retrospective, single-center cohort study. The information was obtained from the database of the CCU of the National Institute of Cardiology in Mexico City. The National Institute of Cardiology is a contemporary reference and teaching center in Mexico City specializing in cardiovascular diseases. For the present study, we analyzed the data from all patients with a STEMI diagnosis admitted consecutively during the period from January 2006 to December 2021. We also identified the patients with CS following the IABP-SHOCK II study definitions [8]: systolic blood pressure < 90 mmHg for ≥ 30 min or the need for catecholamines to maintain systolic blood pressure > 90 mmHg and clinical pulmonary congestion and organ hypoperfusion with any of the following symptoms, cold extremities, confusion or altered mental state, oliguria, or blood lactate ≥ 2.0 mmol/L. Patients who did not have blood pH and lactate measurements on admission were excluded.

In addition, we classified each patient at admission into one of the five classes based on the description of the SCAI and the criteria selected from the Critical Care Cardiology Trials Network Registry [4, 9], and also added the presence of end-organ dysfunction.

### SCAI shock stage A

A patient who is not currently experiencing signs or symptoms of cardiogenic shock but is at risk of its development. These patients may include those with large acute myocardial infarction or prior acute infarction and/or acute or chronic heart failure symptoms without hypoperfusion data.

### SCAI shock stage B

A patient who has clinical evidence of relative hypotension or tachycardia with lactate < 2.0 mmol/L.

### SCAI shock stage C

A patient who on admission meets the CS criteria: systolic blood pressure < 90 mmHg for > 30 min or the need for catecholamines to maintain systolic blood pressure > 90 mmHg, clinical pulmonary congestion, and organ hypoperfusion with blood lactate ≥ 2.0 but < 5.0 mmol/L.

### SCAI shock stage D

A patient who on admission meets the CS criteria and lactate ≤ 5.0 mmol/L or pH ≥ 7.20 and the presence of end-organ dysfunction (liver or kidney) or patients with lactate ≥ 5 mmol/L or pH ≤ 7.20 without the presence of end-organ dysfunction (liver or kidney).

### SCAI shock stage E

A patient who on admission meets the CS criteria and has lactate ≥ 5 mmol/L or pH ≤ 7.20 along with the presence of two dysfunctional end-organs (liver and kidney).

### Definition of end-organ dysfunction

Kidney: abnormal renal function as eGFR < 45 mL/min. Liver: alanine transaminase or aspartate aminotransferase >150 IU/L or three times the upper limit of normal.

The Research and Ethics Committee from the National Institute of Cardiology of Mexico approved this retrospective observational cohort study and was aware that all data were fully anonymized. Patients authorized the use of their clinical data for research purposes with a written informed consent that was obtained upon arrival to the emergency department. All procedures were carried out in accordance with the 2013 Declaration of Helsinki, its addenda, and local regulations. Results will be shared through publications in peer-reviewed journals and presentations at conferences.

### Statistical analysis

All categorical data were summarized as frequencies and percentages. Multiple Kolmogorov–Smirnov tests were applied and indicated that all quantitative study variables were not normally distributed. Continuous variables were reported as medians and 25th and 75th percentiles (interquartile ranges, IQRs). Statistical differences between groups were assessed, either using the chi-square or Fisher’s exact tests in the case of categorical variables or the Kruskal–Wallis test for continuous variables.

The primary study outcome was all-cause mortality at 30 days. The mortality rates were calculated for each SCAI stage and expressed as a percentage; group differences were evaluated by chi-square tests.

Group differences were evaluated by chi-square tests. The discriminatory capacity of the SCAI shock stages for predicting 30-day mortality was determined using the area under the curve (AUC) and discriminative power was determined using C-statistic. Using the cohort of patients with the SCAI stage A as a reference, differences in mortality between the different SCAI shock stages were investigated, survival was plotted with a Kaplan–Meier curve, the patients were censored at the hospital discharge, and differences between groups were assessed by a log-rank test. A multivariable Cox proportional hazards regression model with backward selection was then performed to adjust for potential confounding on the basis of the established associations between each of the SCAI shock stages at admission and the mortality at 30 days. The candidate covariates included in the multivariate analysis were demographic variables, medical history, mechanical complications, and therapies during hospitalization (use of vasopressors, inotropic, intra-aortic balloon pump, and reperfusion therapy). The hazard ratio (HR) with 95% confidence intervals (CI) was calculated. All tests were two-sided and *P* < 0.05 was considered statistically significant. IBM SPSS Statistics for Windows, version 23 (IBM Corp., Armonk, NY, USA) was used.

## Results

**Fig 1:**
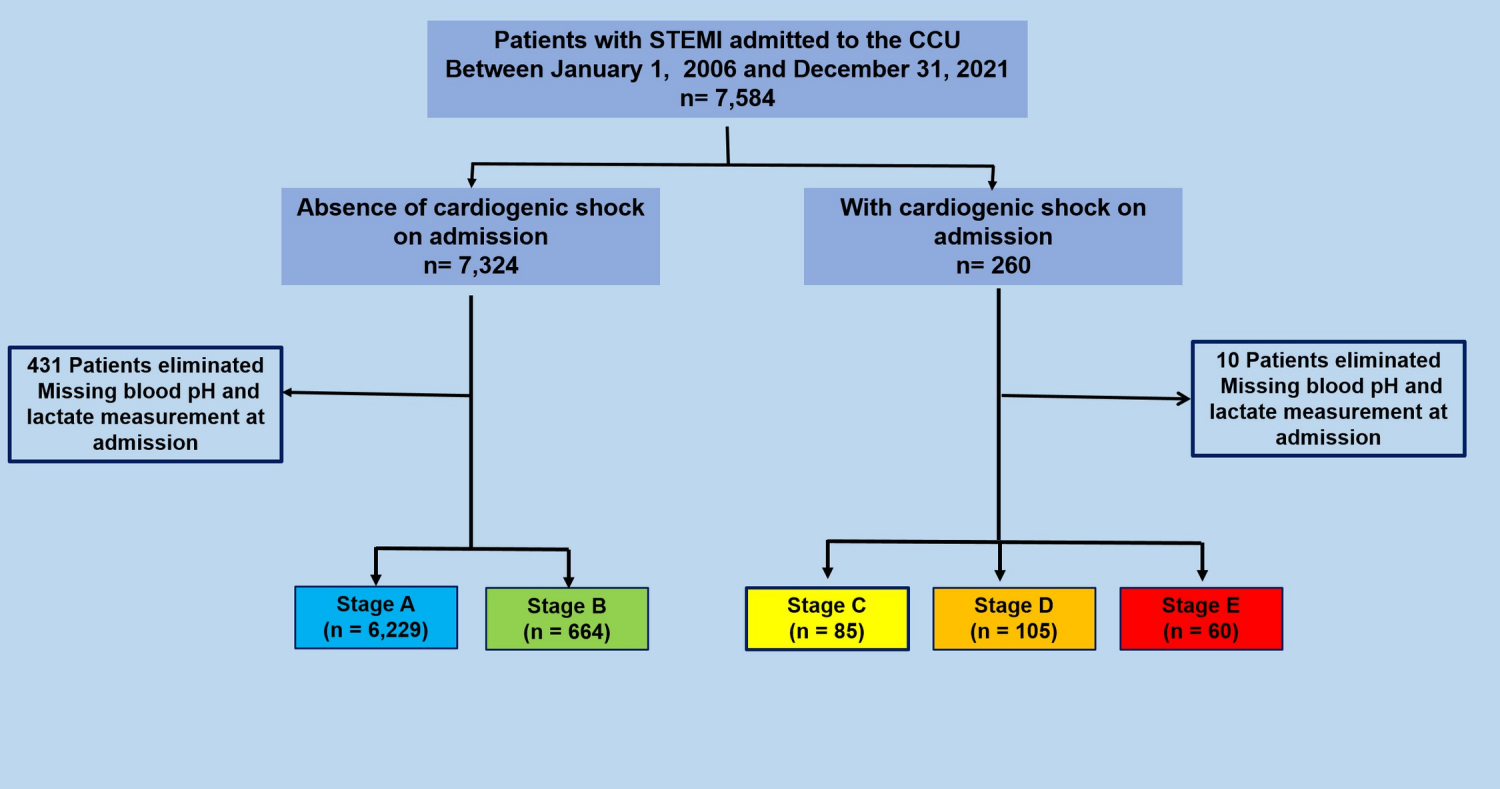
Flow chart illustrating the study sample selection.

During the study period, between January 2006 and December 2021, 7,584 patients were admitted with a diagnosis of STEMI. We excluded 441 patients who did not have pH and lactate measurements at admission. Thus, the study cohort comprised 7,143 patients that fulfilled the inclusion criteria. The patients were classified according to one of the five classes proposed by the SCAI shock stage scheme, with their baseline characteristics at admission as follows: A, 87.5% (6,636); B: 9,1% (688); C, 1.2% (89); D, 1.4% (109); and E, 0.8% (62). (Fig 1). Among the 250 patients with CS at admission, the proportion by stage was as follows: stage C, 34%; stage D, 42%; and stage E, 24%.

Patients with SCAI stages C, D, and E were older, and there was a greater proportion of women. Comorbidities such as diabetes, prior history of myocardial infarction, previous percutaneous coronary intervention, and heart failure were more frequent among patients with SCAI stages C, D, or E (Table 1). Overall, the median time from symptom onset to arrival was 11.17 (IQR, 4.20–37.57) hours. However, in patients with advanced SCAI shock stage (SCAI shock stage C, D, and E), the delay was greater compared with patients in early stages (SCAI shock stages: A and B) (19.37 hours; IQR, 7.41–51.0 vs 11.17 hours; IQR, 4.20–37.57, respectively, *P* < 0.0001).

**Table 1:** Baseline characteristics of patients according to per society for cardiovascular angiography and intervention cardiogenic shock stage.

	SCAI A (n = 6,229)	SCAI B (n = 664)	SCAI C (n = 85)	SCAI D (n = 105)	SCAI E (n = 60)	*P-* Value
**Age median, (IQR) (years)**	59 (52–67)	58 (50–66)	62 (53–70)	65 (54–73)	65 (58–73)	<0.0001
**Female sex, n (%)**	987 (15.8)	114 (17.2)	21 (24.7)	33 (31.4)	14 (23.3)	<0.0001
**Body mass index median, (IQR) (kg/m** ^**2**^ **)**	27.1 (24.8–29.5)	26.7 (24.5–29.3)	27.1 (24.2–31.1)	26.5 (23.8–29.3)	25.9 (24.0–29.3)	0.04
**Medical History**						
**Current smoking, n (%)**	2,176 (34.9)	206 (30.0)	32 (37.6)	28 (26.7)	23 (38.3)	0.10
**Hypertension, n (%)**	2,991 (48.0)	322 (48.5)	42 (49.4)	63 (60.0)	34 (56.7)	0.10
**Dyslipidemia, n (%)**	1,813 (29.1)	183 (27.6)	31 (36.5)	25 (23.8)	13 (21.7)	0.20
**Diabetes, n (%)**	2,247 (36.1)	335 (50.5)	44 (51.8)	60 (57.1)	33 (53.3)	<0.0001
**Previous MI, n (%)**	841 (13.5)	68 (10.2)	17 (20.0)	14 (13.3)	10 (16.7)	0.05
**Previous PCI, n (%)**	484 (7.8)	28 (4.2)	8 (9.4)	5 (4.8)	5 (8.3)	0.01
**Previous heart failure, n (%)**	188 (3.0)	34 (5.1)	5 (5.9)	5 (4.8)	4 (6.7)	0.01
**Previous stroke, n (%)**	126 (2.0)	14 (2.1)	3 (3.5)	3 (2.9)	1 (1.7)	0.85

MI, myocardial infarction; PCI, percutaneous coronary intervention.

At presentation, patients with more advanced SCAI shock stage (C, D, and E) were more likely to have lower mean arterial pressure, no reperfusion therapy, lower LVEF, lower glomerular filtration rates, and higher rates of mechanical complications. (*P* < 0.0001). As expected, laboratory values demonstrated significant end-organ dysfunction in SCAI stages D and E at admission, as evidenced by lactic acidosis, renal impairment, and elevated liver transaminases, which were most pronounced in SCAI stage E (Table 2). Additionally, the use of inotropic agents and vasopressors was higher with the more advanced SCAI shock stage. Less use of intra-aortic balloon pump and invasive ventilation was observed in stage E, compared with stages C and D (Table 3).

**Table 2:** Clinical features and laboratory data at admission of according to per society for cardiovascular angiography and intervention cardiogenic shock stage.

	SCAI A (n = 6,229)	SCAI B (n = 664)	SCAI C (n = 85)	SCAI D (n = 105)	SCAI E (n = 60)	*P-* Value
**Anterior IM, n (%)**	2,22 (46.9)	407 (61.3)	42 (49.4)	56 (53.3)	22 (36.7)	<0.0001
**Systolic blood pressure median, (IQR) (mmHg)**	130 (114–144)	128 (110–149)	86 (80–95)	80 (70–90)	80 (70–84)	<0.0001
**Diastolic blood pressure median, (IQR) (mmHg)**	80 (70–90)	80 (70–90)	50 (40–60)	50 (40–59)	42 (40–50)	<0.0001
**Mean arterial pressure median, (IQR) (mmHg)**	95 (85–107)	96 (83–110)	63 (53–73)	60 (50–67)	55 (50–61)	<0.0001
**Heart rate median, (IQR) (beats/min)**	78 (69–88)	104 (100–110)	100 (78–110)	100 (64–110)	76 (44–109)	<0.0001
**Mechanical complication of CS, n (%)**	125 (2.0)	33 (5.0)	17 (20–0)	19 (18.1)	9 (15.0)	<0.0001
**Lactate, median, (IQR) (mmol/L)**	1.5 (1.1–2.2)	1.3 (1.0–1.6)	2.3 (2.0–3.4)	5.5 (3.1–7.9)	7.35 (5.8–10.1)	<0.0001
**Arterial pH, median, (IQR)**	7.41 (7.38–7.45)	7.42 (7.38–7.46)	7.37 (7.29–7.43)	7.28 (7.21–7.31)	7.18 (7.09–7.27)	<0.0001
**Bicarbonate, median, (IQR) (mEq/L)**	20.4 (18.6–22.5)	21.0 (19.0–22.9)	17.9 (15.4–20.8)	15.0 (12.0–17.0)	12.8 (10.0–14.5)	<0.0001
*Glomerular filtration rate, median, (IQR) (**mL/min)**	84.7 (63.0–107.2)	82.6 (59.5–111.1)	56.7 (35.9–74.5)	36.2 (27.8–55.0)	27.0 (20.9–33.7)	<0.0001
**Alanine aminotransferase, median, (IQR), (U/L)**	84 (38–198)	75 (36–176)	126 (41–322)	113 (74–380)	700 (379–1427)	<0.0001
**Aspartate aminotransferase, median, (IQR), (U/L)**	47 (30–76)	45 (28–73)	58 (31–125)	324 (126–699)	424 (143–1610)	<0.0001
**LVEF median, (IQR) (%)**	50 (40–55)	43 (33–52)	33 (28–40)	30 (20–39)	30 (20–40)	<0.0001
**Creatine kinase-MB, median, (IQR) (mg/mL)**	27.0 (5.9–109.0)	21.9 (5.5–79.5)	22.6 (5.6–181.1)	74.0 (27.202.0)	110 (260–259.0)	<0.0001

MI, myocardial infarction; LVEF, Left ventricular ejection fraction;

* Glomerular filtration rate according to the Cockroft-Gault formula

**Table 3:** Therapies during hospitalization of according to society for cardiovascular angiography and intervention cardiogenic shock stage.

	SCAI A (n = 6,229)	SCAI B (n = 664)	SCAI C (n = 85)	SCAI D (n = 105)	SCAI E (n = 60)	*P-* Value
**No reperfusion therapy, n (%)**	2,218 (35.6)	327 (49.2)	47 (55.3)	51 (48.6)	36 (60.0)	<0.0001
**Primary PCI, n (%)**	2,227 (35.8)	179 (27.0)	19 (22.4)	31 (29.5)	13 (21.7)	<0.0001
**Thrombolysis (%)**	1,784 (28.6)	158 (23.8)	19 (22.4)	23 (21.9)	11 (18.3)	<0.0001
**Inotropes IV, n (%)**	451 (7.2%)	176 (11.4)	71 (83.5)	83 (79.0)	51 (85.0)	<0.0001
**Vasopressors IV, n (%)**	612 (9.8)	88 (13.3)	80 (94.1)	96 (94.1)	58 (96.7)	<0.0001
**IABP, n (%)**	237 (3.8)	41 (16.2)	45 (52.9)	47 (44.8)	19 (31.7)	<0.0001
**Mechanical ventilation, n (%)**	348 (5.6)	59 (8.9)	62 (72.9)	83 (79.0)	44 (73.3)	<0.0001

**Fig 2:**
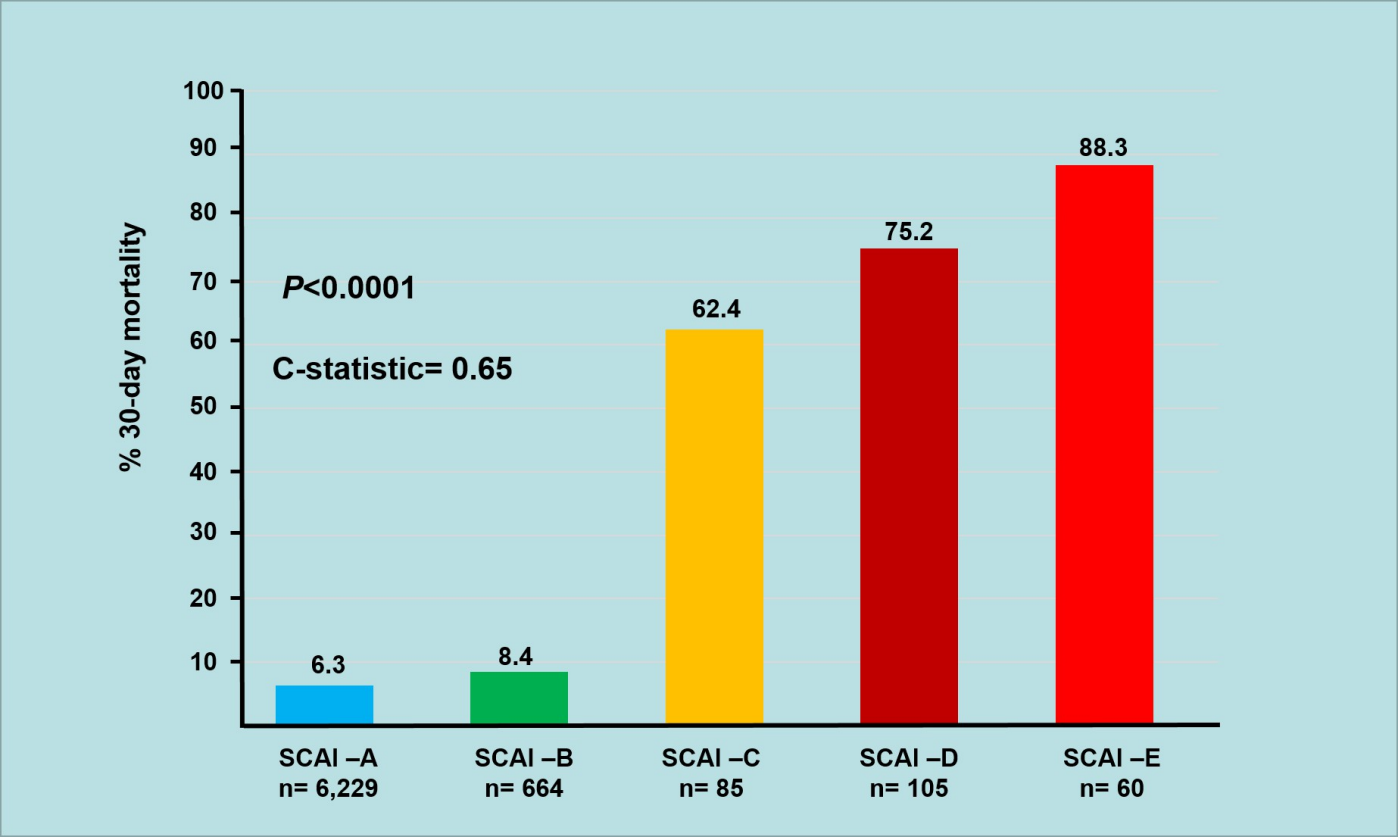
SCAI shock stages with corresponding 30-day mortality rate and discriminatory performance.

**Fig 3:**
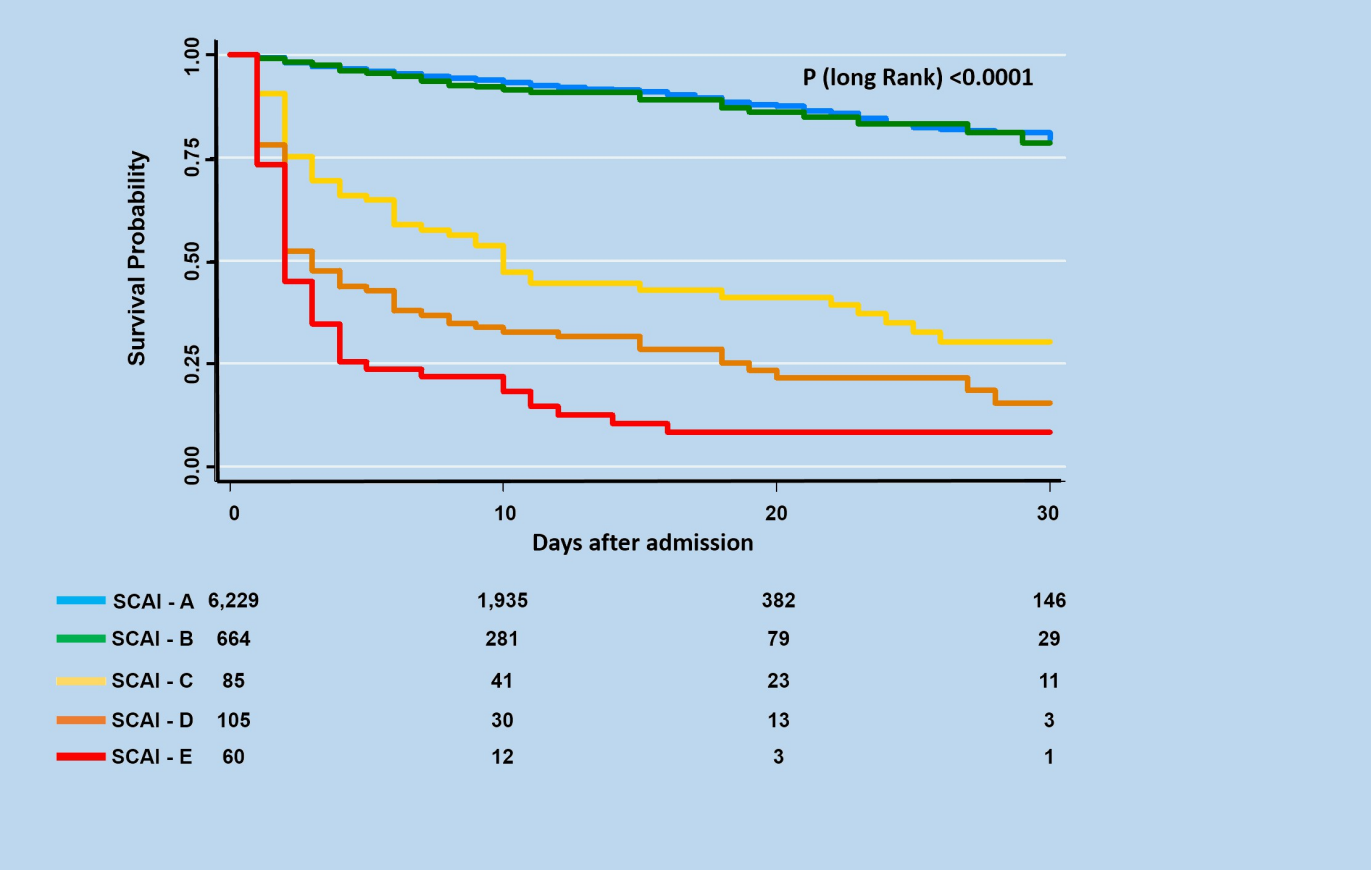
Kaplan-Meier survival curves for 30-day hospital survivors as a function of SCAI shock stage.

**Fig 4:**
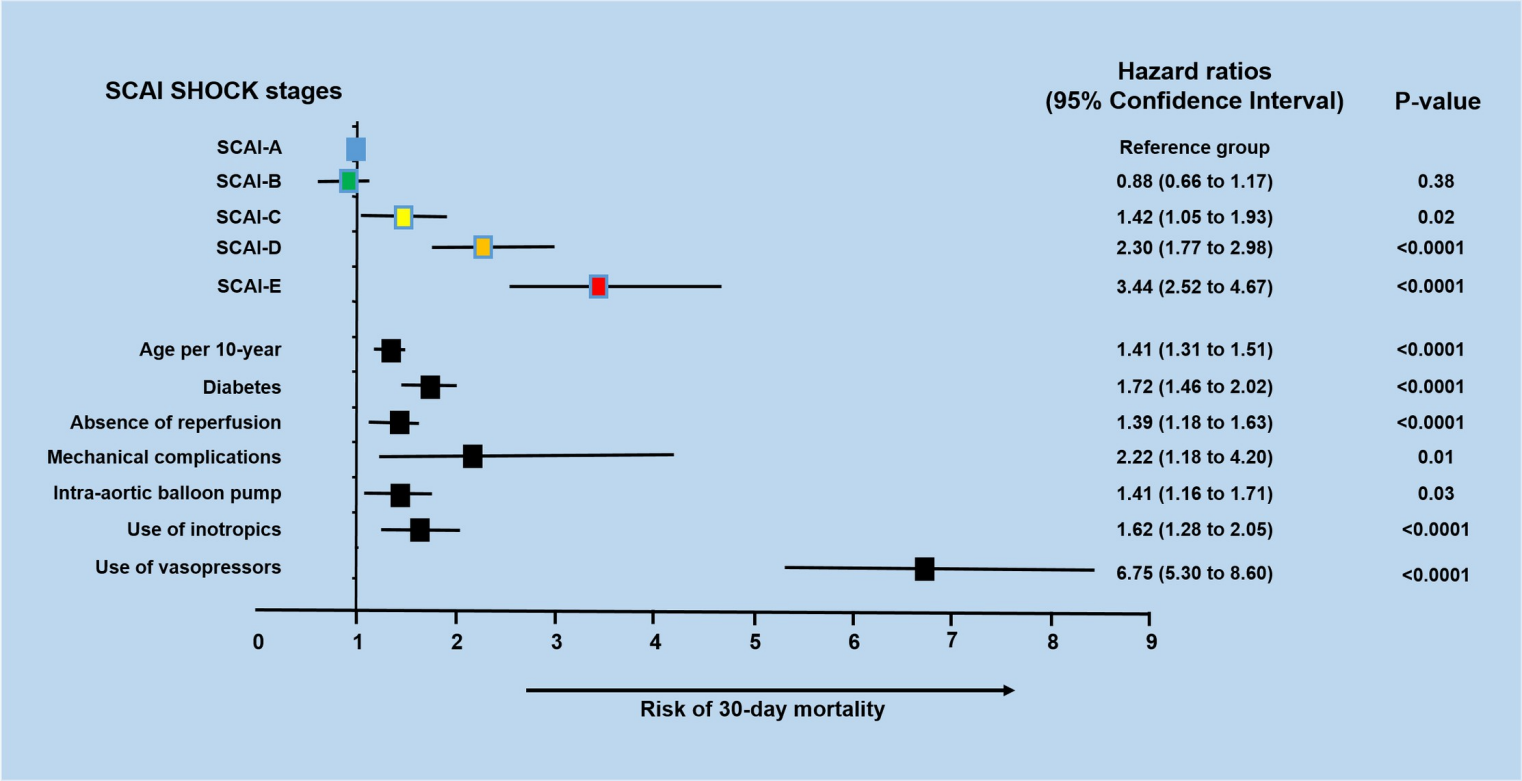
Adjusted hazard ratio for 30-day mortality demonstrating adjusted associations between SCAI shock stages with 30-day mortality.

PCI, percutaneous coronary intervention, IABP, intra-aortic balloon pump;

During the 30-day follow-up, mortality occurred in 632 (8.8%) of the 7,143 STEMI patients included in the study. The unadjusted 30-day mortality rates increased progressively with each SCAI shock stage (6.3%, 8.4%, 62.4%, 75.2%, and 88.3% for A, B, C, D, and E stages, respectively; *P* < 0.0001). Admission SCAI shock stage had good discriminatory power for 30-day mortality (AUC, 0.65; 95% CI, 0.62–0.67) (Fig 2). A trend toward lower 30-day survival probability was observed in patients classified with advanced CS (30.3, 15.4%, and 8.3%, SCAI shock stages C, D, and E, respectively) (Fig 3). A striking result to emerge from the data is that of the deaths recorded in the groups of patients with advanced CS, around 70% of them occurred very early (within the first 72 hours) (49.0%, 69.6%, and 73.5% in SCAI shock stages C, D, and E, respectively). After adjusting for potential confounders in the multivariate analysis, using SCAI shock stage A as a reference, the higher SCAI shock stages remained an independent mortality risk with Cox proportional HRs of 1.42 (95% CI 1.05–1.93, *P* = 0.02), 2.30 (95% CI 1.77–2.89, *P* < 0.0001), and 3.44 (95% CI 2.52–4.67, P < 0.0001) for the C, D, and E SCAI shock stages, respectively (Fig 4).

## Discussion

In a retrospective cohort of patients with STEMI, we applied the SCAI shock stage classification at the time of hospital admission. Among the findings of this study, first, we found that the application of the SCAI scheme in a real-world population with STEMI identifies very high-risk patients at hospital admission. Second, there was a stepwise increase in mortality across the SCAI shock stages; their application showed good discrimination for 30-day mortality. Third, after adjusting for potential confounders in the multivariate analysis, the advanced SCAI shock stages (D and E) remained with an up to 3.44 times greater risk of mortality. Finally, of the deaths registered in patients with shock stages D and E, around 70% occurred in the first 72 hours after admission.

With the aim of improving CS patients’ risk stratification, in 2019 the SCAI proposed a new classification to describe CS severity and facilitate patient triage between providers in different care settings, and select advanced therapies [4]. Despite each study using different criteria to define the SCAI stages, SCAI shock staging criteria have been applied and validated in several large retrospective clinical cohorts, consistently demonstrating an association between SCAI shock stage and risk of mortality in a variety of populations [5–7, 9–13], such that classification of shock severity is feasible in research using definitions that could be applied in clinical practice and provides robust risk stratification of CS patients [14].

Jentzer et al. [5] from the Mayo Clinic retrospectively applied the SCAI shock stages in patients with AMI-CS and those with CS due to heart failure in the first 24 hours after admission to the intensive care unit, demonstrated that the hospital mortality varied from 3% in stage A to 67% in stage E, and shock severity as measured using the SCAI stages was a strong predictor of mortality even after adjusting for standard clinical risk factor.

In the National Cardiogenic Shock Initiative database the SCAI shock stages were applied retrospectively to 300 patients who presented AMI-CS and needed MCS therapy, patients were categorized as SCAI stages C–E in the first 24 hours. The survival to hospital discharge was seen in 76% of patients assigned to stage C or D, compared to those in stage E (58%; p = 0.006). Additionally, in patients who were initially assigned a lower SCAI stage (C or D) and who deteriorated to stage E over 24 hours, <20% survived to hospital discharge [6]. Similar results had been reported by Schrage et al. [7] in Germany on 1007 consecutive patients with CS-AMI; the 30-day survival was 96.4% for those in SCAI Stage A, 46.1% in Stage C, and only 22.6% in stage E.

The present study was designed to determine the effect of the SCAI shock stage classification with the information obtained at admission in a cohort of non-selected patients with STEMI from a reference tertiary care center in a middle-income country. Pragmatically, the SCAI shock stage classification is a dynamic process that requires information during the evolution of decision-making regarding the next level of care and therapy. However, most studies have classified the SCAI shock stage at a single time point (generally 24 hours after admission), precluding an analysis of serial changes in stage over time. Notably, in most of the studies, the stage is assigned retrospectively, taking into account various combinations of clinical variables based on the availability of data [5–7, 9–13]. We classified our population studied at admission, and added the presence of end-organ dysfunction (kidney and/or liver), since stages D and E represent a deficiency of response to initial management, characterized by rising lactate, worsening of pH, and/or increasing vasopressor or mechanical circulatory support requirements, and the presence of organ failure.

Our results show that the overall 30-day mortality for all cohort of patients with advanced CS (SCAI shock stages C, D, and E) was substantially higher (74%), which is in stark contrast to rates reported in the US and European registries of 50% [15, 16]. These differences can be explained in part by differences in demographic, clinical, and comorbidity characteristics compared with the large registries from the United States and Europe, as well as the patients’ delayed presentation at hospital admission after first experiencing AMI symptoms and a higher rate of patients who did not receive reperfusion therapy, which is common in low- and middle-income countries [17].

This study produced results that are significant in the following respects. Our results are consistent with data obtained in previous studies, indicating that mortality was substantially higher among patients with SCAI stages D and E (75.2% and 88.3%, respectively), who were identified as having advanced hemodynamic and metabolic shock states at admission (lactic acidosis, renal impairment, and liver injury). The application of the SCAI shock stage to our study population showed high discrimination for 30-day mortality, similar to that reported by Jentzer et al. (AUC, 0.65 vs 0.68) [18]. Another important finding is the very high mortality (70%) in the first 72 hours after admission in patients with shock stages D and E.

The primary data available on the application of the SCAI shock stages classification come from registries of cardiovascular intensive care units in high-income countries with access to advanced therapies. Cardiogenic shock is an area of burgeoning interest also in low- and middle-income countries, reflecting the demographic changes and the epidemiological transition to cardiovascular diseases occurring in the latter countries. These findings can help us understand the characteristics and outcomes of patients classified by SCAI shock stages in a coronary care unit in a middle-income country. If corroborated by other studies, these findings may have profound clinical implications for the contemporary management of CS. The advent of risk stratification based on hemodynamic and metabolic status, and end-organ dysfunction raises new hopes regarding improved prognostic algorithms in CS.

In developing countries such as Mexico, unfavorable social circumstances and inadequate and inefficient public spending on health care can present considerable barriers to improving outcomes in patients with CS. The diffusion of the application of the SCAI classification can be a tool to identify in timely manner patients at high risk for developing CS or with impending CS. They will likely need a higher level of care, where multidisciplinary models involve CS teams, structured referral schemes, and standardized cardiovascular intensive care units. Mexican researchers in this field must start collaboration programs between managing patients with capable CS centers and specialized tertiary hospitals with multidisciplinary CS teams, according to the ’hub and spoke’ model, to coordinate the most appropriate and time-effective therapeutic strategy.

### Study limitations

Our trial has several limitations. First, our study was retrospective and reflected the experiences of a single tertiary center specializing in cardiovascular diseases. The second limitation relates to the study design. We did not use a pragmatic application of the criteria of the SCAI cardiogenic shock staging. Third, we only analyzed information at admission; our database did not allow the analysis of initiation and changes in doses of vasopressors and inotropes, serial information of various laboratory data, including blood gases, or time of installation of BIAC. Thus, larger prospective studies will need to be performed to confirm these results.

## Conclusions

This analysis of 7,143 patients with STEMI admitted to a university tertiary center in Mexico showed the SCAI shock stages applied to admission despite different criteria, populations and therapies remained consistent as a predictor of 30-day mortality, so it is a useful tool for risk stratification when admitting patients across the full spectrum of CS. If corroborated by other studies, these findings may have profound clinical implications for the contemporary management of CS. The advent of risk stratification based on hemodynamic and metabolic status, and end-organ dysfunction raises new hopes regarding improved prognostic algorithms in CS.
